# Citizens’ initiatives for care and welfare in the Netherlands: an ecological analysis

**DOI:** 10.1186/s12889-019-7599-y

**Published:** 2019-10-22

**Authors:** Thijs van der Knaap, Jan Smelik, Floor de Jong, Peter Spreeuwenberg, Peter P. Groenewegen

**Affiliations:** 1 Department of Sociology, Utrecht University, and intern at Nivel – Netherlands Institute for Health Services Research, Nivel, PO Box 1568, 3500BN Utrecht, The Netherlands; 2 Nederland Zorgt Voor Elkaar, Amersfoortseweg 38, 3951 LC Maarn, The Netherlands; 3 Vilans – Centre of Expertise for Long-term Care, PO Box 8228, 3503RE Utrecht, The Netherlands; 4 Nivel – Netherlands Institute for Health Services Research, Nivel, PO Box 1568, 3500BN Utrecht, The Netherlands; 5 Nivel – Netherlands Institute for Health Services Research, and Department of Sociology and Department of Human Geography Utrecht University, Nivel, PO Box 1568, 3500BN Utrecht, The Netherlands

**Keywords:** Citizens’ initiatives, Social cooperatives, Geographical distribution, Social capital, Ecological analysis, The Netherlands

## Abstract

**Background:**

In the Netherlands as well as in other countries citizens take initiatives to provide or maintain services in the area of care and welfare. Citizens’ initiatives (CI’s) are organisations some of which have a formal structure while others are informally connected groups of citizens, that are established by a group of citizens with the aim to increase the health and welfare within their local community and that are not focused on making a profit. Although CI’s have been around since at least the 1970’s little research has been done on the phenomenon, with most of it consisting of case studies or qualitative exploratory research. To fill part of this gap in knowledge, we have studied the geographical variation in the presence of CI’s in the Netherlands and tried to explain this variation.

**Methods:**

Data on the presence of CI’s were obtained by combining two existing inventories. We did an ecological regression analysis to test hypotheses about the relationship between the presence of CI’s and the existence of a care vacuum, the capacity for self-organisation and models of action in local communities.

**Results:**

We counted 452 CI’s in care and welfare in the Netherlands in January 2016. Our results show a spatial concentration of care initiatives in urban areas in the Randstad cities in the west of the country and in rural areas in the south-east. The presence of CI’s is only weakly associated with a care vacuum, but is related to indicators for the capacity of concerted action and models of action.

**Conclusion:**

There are by now a considerable number of CI’s in the area of care and welfare in the Netherlands. Apparently, citizens take collective initiatives to provide services that are not, or no longer, available to the local community. The initiatives are concentrated in certain parts of the country. However, our theoretical model to explain this geographical pattern is only partially confirmed.

## Background

Citizens initiatives (CI’s) are an international phenomenon and present in multiple countries in Europe and North-America. Italy is one of the earliest examples of a country where in modern times CI’s have started that provide a significant amount of care and welfare [5, 41, 44]. Geographically speaking social cooperatives (the broader term used in Italy) are more prevalent and successful in the north of the country compared to the middle and south of Italy and the Italian islands [8]. The geographical pattern has not been further explored, but it might be related to lower levels of social trust and civic engagement in the south [31].

In the UK some research has been done on the geographic distribution of social enterprises (which comes close to CI’s in the Netherlands), based on case studies [7]. Amin [2, 7] suggested conditions that favour the emergence and success of social enterprises, among these the support of local authorities, the presence of other associations, network resources and the extent of social deprivation. Buckingham et al. [7] hypothesized the importance of local need and the capacity to organise social enterprises, such as social capital and government support in deprived areas that lack social capital. They used existing data to examine regional differences in the presence of social enterprises; however, differences in definitions and measurements make these data largely incomparable. They conclude that social enterprises seem to be fairly evenly distributed over the UK, but that more variation might be found within cities and in smaller areas.

In the United States the so-called ‘Village model’ provides services in the area of welfare, by developing social support and engagement to enable older people to age in place. Scharlach et al. [35] define ‘Villages’ as follows: ‘Villages are membership associations developed and operated by older community members for the primary purpose of enhancing their quality of life and ability to age in place.’ (p. 183). Their survey shows that ‘villages’ often also provide care services, such as chronic disease self-management education. Davitt et al. [9] mention a number of key factors that might be related to the emergence of ‘Villages’. Among these are what the authors call policy gaps, resulting from the focus of current policies for elderly on specific needs, on the most vulnerable groups, or on low income elderly, leaving large groups that fall outside the eligibility criteria without support. They also mention a lack of age-friendly communities, of informal support, of social ties, and of involvement of elderly people themselves in the planning and development of services. They stress the importance of community capacity for self-organisation. However, they do not analyse the geographical distribution of ‘Villages’ in the light of these key factors.

In the Netherlands the ‘movement’ of citizens initiatives that provide care and welfare started much later, in 2005 when an initiative started in Hoogeloon in the south of the Netherlands. Since then it has grown into a movement with at least 450 initiatives nationwide in 2016. At the same time the national government made a major policy reform of which a central idea was that people in need of care should firstly rely on their social network and that the community should be more responsible for care and welfare. This idea has taken hold in Dutch politics and is laid down in legislation, starting from the 2007 Social Support Act that was extended in 2015 and the Participation Act and the Youth Act of 2015 [23, 27]. This legislation devolved responsibilities in the area of care and welfare from the central government to the municipalities. One of the reasons for decentralization was the idea that municipalities are better able to provide tailor-made care and that this would increase quality [26]. With the devolvement of responsibilities to municipalities came budget cuts that made it more pressing for municipalities to rely on informal support and care.

There are many different forms of CI’s in care and welfare. We base our definition on previous inventories in the Netherlands ([42]; De [10]) and the international definition by Spear [39]. Based on these studies, we come to the following definition for CI’s in care and welfare: *organisations that are established by a group of citizens with the aim to increase the health and welfare within the local community in which the organisation operates and that are not focused on making a profit.* The term ‘organisation’ is not used in the sense of a formal organisation; CI’s may be informal groupings or networks of people that are goal-oriented and coordinate actions.

Against this background we want to answer the following research questions:

*What is the geographical distribution of CI’s in care and welfare in the Netherlands?*

*How can the geographical distribution of CI’s in care and welfare in the Netherlands be explained?*

### Theoretical background and hypotheses

CI’s are local phenomena. We use small geographical areas (see data and methods section) to indicate local communities. These communities either have or do not have one or more CI’s. The communities also have other characteristics that might explain why there is an initiative in some and not in others. Here we will develop hypotheses about these characteristics.

CI’s are the result of concerted actions of individuals. Hypotheses at community level about the presence of initiatives are therefore based on assumptions about actions of individuals, combined with conditions that favour concerted action at community level. We assume three basic mechanisms [17]. The first is that individual actors must have a feeling that something has to be done to compensate for lack of services in the area of health and welfare or preserve them for the future. This lack of service we will call a care vacuum. A care vacuum is a situation in which neither the government nor the market provides health and welfare services in a way that fits with the needs of the community. Moreover, there must be at least a number of individuals in the community that share this *feeling of necessity*. The second mechanism is a concerted action generating mechanism which is at community level: the *capacity for collaboration*. Finally, it helps when people who feel the necessity for action and have the capacity to collaborate, have access to examples of CI’s or ways of organising themselves in general; in other words: when there are *models of action*.

#### Hypothesis 1

*In communities where a care vacuum exists, CI’s in care and welfare are more likely to be present.*

We expect a care vacuum to exist if:

- demand for certain services is higher due to an older population; older people increasingly wish to age in place and current policies stimulate this. The need for care and support services is higher with an older population. If regional variation in ageing of the population is not matched by supply of care and support services, a care vacuum develops.
- supply of care or welfare services is lower due to relocation or concentration of services leading to larger distances; there is a general trend towards larger organisations which expresses itself e.g. in fewer and larger municipalities in the Netherlands and larger general practices (from 56% in single-handed practice in 2001 to 18% in 2016) [21, 43]. Traveling to health care services can be difficult, especially for vulnerable people that need them most, which aggravates the problem [11].
- there is a mismatch of supply and demand due to diverse preferences by ethnicity; people with a migration background have other preferences compared to the general population [12, 16, 18].

In addition, we expect that a care vacuum may also be experienced when general services, such as schools or shops, are farther away or have disappeared from the community. When general services disappear, this often triggers a strong reaction from citizens. This can be a catalyst for action both to replace general services and start services in the sphere of care and welfare.

#### Hypothesis 2

*In communities with more capacity for concerted action, CI’s are more likely to be present.*

We expect more capacity to exist if:

- the community has more social capital; social capital refers to the resources that reside in the connections between people and the networks that enhance mutual trust and cooperation [24, 29]. Communities with more social capital will be more likely to engage in collective action and have lower transaction and monitoring costs. Moreover, when people in the community interact, the likelihood increases to identify that they share a definition of their situation as needing change.
- population turnover is lower; high residential turnover disrupts existing relationships and weakens interpersonal ties and discourages investment in relationships (social capital) in one’s neighborhood [15, 34]. Furthermore, high residential turnover can prevent a community to achieve a shared feeling of necessity of action because the perspective on problems of new inhabitants may differ from the old inhabitants.
- there are more people with time to do voluntary work; this will be operationalized through the percentage of people aged 65 to 75. The decline of mandatory activities enables more people in this age range to undertake non-work activities and a large number uses some of this time to perform voluntary work [47], aimed at care [3]. People aged 65 to 75 often still have the capacity to do this, due to an increasing life expectancy without moderate or serious illness [40].
- there are more higher educated people; higher educated people are more likely to volunteer [3, 37] because they have a higher level of empathy, are more self-confident [6, 33] and have a stronger sense of civic responsibility [30].
- there are more people with a religious affiliation. They are more altruistic and prone to voluntary giving and pro-social behavior [4, 36]. The effect of religion differs between different faiths. Protestants are more likely to organise themselves in smaller exclusive groups compared to Catholics. This is because of their stricter ways of practicing their religion and higher level of theological exclusivity [20, 25]. This means that the emergence of CI’s is hypothesized to be more likely in areas with many Catholics.

#### Hypothesis 3

*In communities where models of action are available, CI’s are more likely to be present.*

We expect models of action to be available if:

- other CI’s are nearby; people aspiring to start an organisation look around and share, take over or imitate ideas [1].

Apart from these main effects, we hypothesize that the combination (or interaction effect of the three mechanisms) would make it most likely that CI’s are present.

## Data and methods

### Inventory of CI’s

CI’s are a relatively new phenomenon in the Netherlands. They differ in size, focus, organisational form etc. It was therefore difficult to find a complete inventory of all initiatives. We found 452 initiatives, active on the 1st of January 2016. We came to this number by merging two existing inventories and testing the merged dataset for completeness. After that, the resulting dataset was extended with help of ‘*Nederland zorgt voor elkaar’,* a national network of CI’s in care and welfare that was established in 2016.

Initiatives are considered active if they have given a “sign of life” after the 1st of January 2016. This sign can be a post on their website, a post on social media, a piece in a (local) paper or other news source. They are considered stopped when there is evidence that they stopped all activities; this can be in the form of an online post, newsletter, local news source, etc. Not active are the initiatives that have not given a sign of life after January 2016 but also have not given evidence that they stopped.

The first inventory we used was completed in August 2016 by Vilans and includes approximately 320 initiatives (De [10]). The second inventory was completed in July 2016 by the research group ‘Institutions for collective action’ and includes 240 initiatives.

These inventories have been combined into one more complete dataset. Because of the way information for these inventories has been collected, they may be incomplete. Two tests have been done to find out how complete the merged datasets of citizens initiatives are. The first was checking what percentage of initiatives that are part of the merged dataset (*n* = 230) are also found on the internet. 58% (134/230) were found on the internet. This percentage is high considering the fact that CI’s are often informal organisations and are locally oriented. Hence, we may assume that they are not always interested in online presence because most contact is face to face. Furthermore, the target group for many of the CI’s is elderly people who are less active on the internet. The second test for completeness of the dataset was to assess how many of new initiatives are found on the internet that were not in the dataset. This may indicate regional bias in the dataset. Seventeen new active initiatives were found that were not yet part of the merged dataset. Six of these were based in the province of Friesland that only had three initiatives in the merged dataset. This might indicate regional selectivity in the completeness of the dataset. The fact that we only found 17 new initiatives might indicate again that CI’s are hard to find via the internet.

We therefore sought the help of the umbrella association of CI’s, ‘*Nederland zorgt voor elkaar’*. Using their data we were able to extent the inventory with another 222 initiatives, leading to a full dataset consisting of 452 initiatives that we think were active on 1 January 2016.

### Geographic unit of analysis

Based on the hypotheses, the ideal geographic unit to study the presence of CI’s consists of areas that form communities [7, 45]. recommended to use small areas. In particular the capacity mechanism of hypothesis 2 presupposes a scale at which people know each other and where they might share a feeling of necessity. In urban areas, this is the scale of city neighbourhoods and in rural areas small villages or settlements. We have used four-digit postal code areas because these come closest to this. Moreover, data are available at that scale to operationalise our hypotheses. Their surface varies between 1 and 8 km^2^, with an average population of 4160 (interquartile range 6140). In urban areas, they are relatively small, with a higher number of inhabitants and consequently denser population; in urban areas postal code areas largely coincide with urban neighbourhoods. In rural areas, whole villages or settlements and the surrounding area belong to the same postal code area.

### Independent variables

To test hypothesis 1 on the existence of a care vacuum, the following characteristics of communities were used:

- Percentage of people aged 75 or over, source: Statistics Netherlands, 2010.
- Percentage of people with a migration background, source: Statistics Netherlands, 2010.

The other independent variables are from Statistics Netherlands, 2015.

- Linear distance in kilometers to three different care services: general practitioners, pharmacies and hospitals.
- Linear distance in kilometers to four different general services: a store for daily groceries, elementary school, high school and library.
- Increase in distance to care services between 2008 and 2015 (0 = no increase).
- Increase in distance to general services between 2008 and 2015 (0 = no increase).

To test hypothesis 2 on the capacity for concerted action, the following characteristics of communities were used:

- Social capital; source: constructed on the basis of the ‘Housing and Living Survey 2012’, made available by Statistics Netherlands. Statistics Netherlands gave access to data of postal code areas with at least three respondents, resulting in 2544 areas with on average 27 respondents. The social capital measure was constructed, using an ecometric analysis ([32]; following [28] and [46]), from five questions on contacts with direct neighbours; contact with other neighbours; whether people in the neighbourhood know each other; whether neighbours are friendly to each other; and whether there is a friendly and sociable atmosphere in the neighbourhood.
- Percentage of people aged 65 to 75, as operationalization of availability of people with time to do voluntary work; source: Statistics Netherlands, for the year 2010.

The other variables are from Statistics Netherlands, 2015 on the municipal level:

- Percentage of people that have completed higher education (university of applied science or university).
- Percentage of people with Catholic religious affiliation.
- Population turnover as proportion of the population.

To test hypothesis 3 on models of action we used the shortest (linear) distance from each postal code area to a postal code area with a CI.

We used three potential confounders:

- Level of urbanization; source: Statistics Netherlands, for the year 2012 on postal code area level, in five categories ranging from very strongly urban (1) to non-urban (5), based on the number of households per square kilometre, which is widely used in the Netherlands [14].
- Population size; source: Statistics Netherlands, for the year 2010 on postal code area level.
- Socio-economic status; source: Statistics Netherlands for the year 2014. This variable is based on average income, the percentage of people with a low income, the percentage of people with low education and the percentage of non-working people [22].

### Statistical analysis

The descriptive research question is answered by a visual inspection of the map of the Netherlands. This shows the geographical pattern. We have further quantified this by assessing the clustering of CI’s within provinces and municipalities.

For the explanatory analysis, the dependent variable is whether or not one or more CI’s are present in a four-digit postal code area. We therefore used logistic regression analysis. The units of analysis – the four-digit postal code areas – are nested within municipalities and provinces. The municipalities play a role in the provision of care and welfare services, following the devolvement of responsibilities in Dutch policies. They are consequently a relevant context for the presence of CI’s. Moreover, some independent variables were only available at municipal level. We added the level of the provinces to show the clustering of CI’s. To take this into account we did a three-level multilevel analysis with the postal code areas at the lowest level, the municipalities at the intermediate level and the provinces at the highest level. All continuous independent variables were centred.

As a modelling strategy we took the following steps: We first estimated the empty model for all postal code areas. This only models the average and the variances. The variances at municipal and provincial level are an indication for geographical clustering of CI’s. We then subsequently added the confounders, then the variables to indicate a care vacuum, then the capacity variables and finally the models of action variable. As the stepwise modelling strategy did not add much information, we decided to only present the empty model and the model with all independent variables.

We then proceeded with a stratified analysis according to level of urbanization (in three categories). The reason is that CI’s in rural areas, with in general a lower level of services of all kinds, might be associated with different variables compared to the ones in urban areas. Moreover, visual inspection of the spatial distribution of CI’s and the analysis over all postal code areas showed that CI’s are more prevalent in cities and rural areas and less so in areas of intermediate level of urbanization. We again followed a stepwise approach and only present the full model. As an approximation of the explanatory power of these models we have added McKelvey and Zavoina’s pseudo R-squared, including random and fixed effects and fixed effects only, following Snijders and Bosker [38].

Our theoretical model assumes interaction effects of the existence of a care vacuum, the capacity variables and the models of action variable. Because of the large number of separate indicators of a care vacuum, the number of interaction terms would become very high. We therefore decided to reduce the number of independent variables. We only included independent variables, when the standard error was smaller than the regression coefficient (equivalent to *p* < .30) [19]. After this we estimated interactions of variables with a significant main effect in the group of care vacuum variables, capacity variables and models of action variable. We did this both for all postal code areas and for the stratified analysis.

All analyses were done in MLwiN 3.0.

## Results

### Spatial distribution of CI’s

**Fig. 1:**
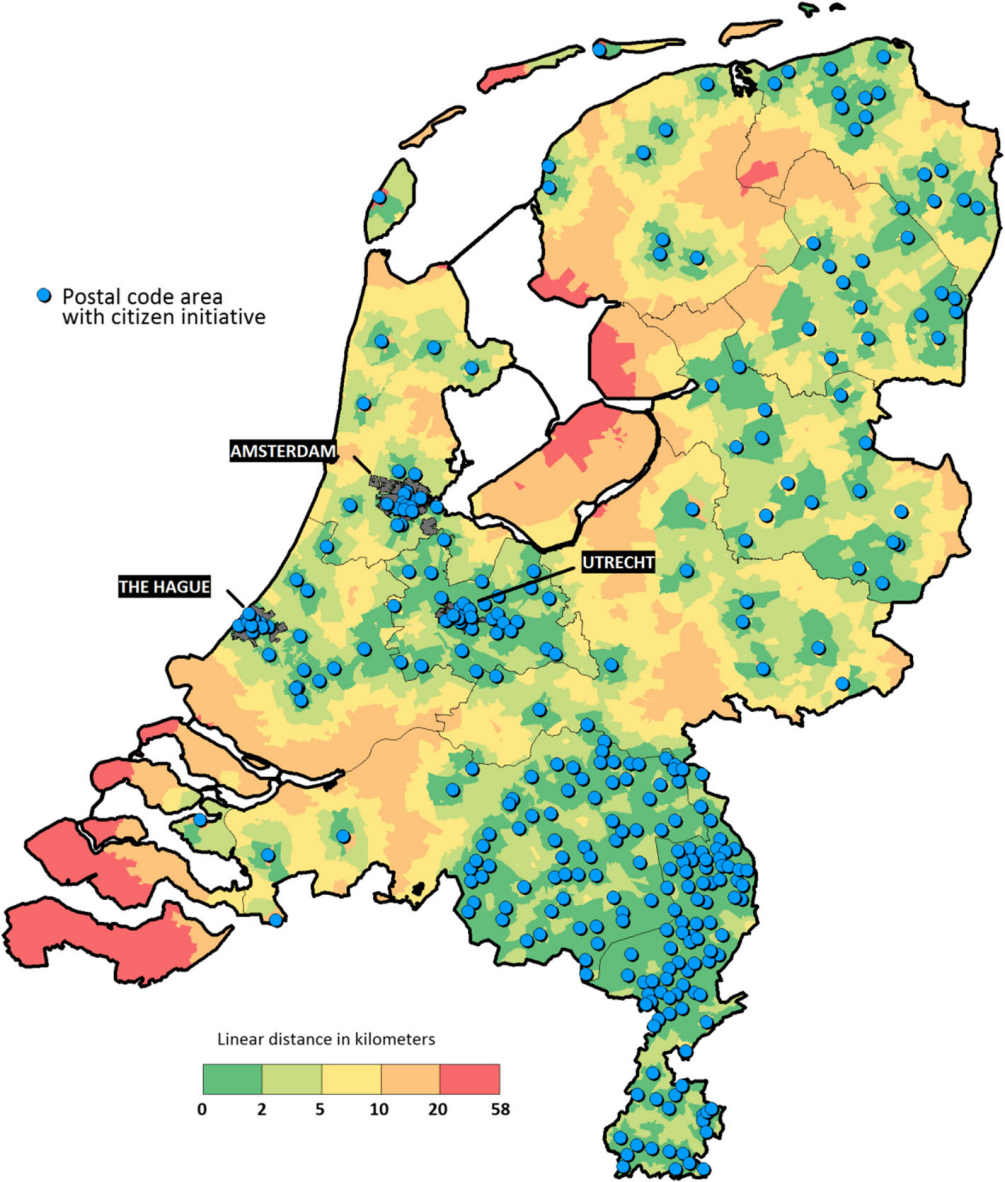
Shortest (linear) distance from each postal code area to a postal code area with a CI (2016). Map of the Netherlands including province boundaries. Postal code areas with a CI and shortest linear distance from each postal code area to a postal code area with a CI. Data for presence of active CI’s at 1 January 2016. Source: own production

We counted 452 CI’s in the country. Most CI’s are active in the south-east of the country, the provinces of Noord-Brabant and Zuid-Limburg (Fig. 1). The provinces of Flevoland (no initiatives) and Zeeland (one initiative) have fewest. When related to population size, the picture is only slightly different with Zuid-Limburg having the highest density of CI’s, followed by the province of Groningen in the north-east and the province of Noord-Brabant (not in table). CI’s are more often present in urban postal code areas and rural areas and less so in intermediate categories of urbanisation. This is to a large extend due to the Randstad provinces Noord- and Zuid-Holland and Utrecht where CI’s are mostly active around and in the cities Amsterdam, The Hague and Utrecht. In all other provinces (except for Friesland in the north-east) CI’s are in varying degrees more active in rural postal code areas.

The statistical analysis shows significant variances at province and municipal level (Table 1). This illustrates again the spatial clustering of CI’s. The intra-class correlations in the empty model are reasonably high for all postal code areas; 25% at municipal level and 24% at province level. For the subgroup of semi-rural and rural areas the intra-class correlations are 21 and 35% respectively. This indicates that CI’s in (semi-)rural areas are strongly clustered in provinces.

**Table 1:** Variances and intra-class correlations from a logistic multilevel analysis with dependent variable presence (1) or absence (0) of a citizen’s initiative in the empty model

	All postal code areas	Urban ^a^	Semi-urban and intermediate	Semi-rural and rural
Variance municipality	1.652^*^	1.004^*^	0.680^*^	1.545^*^
Variance province	1.598^*^	–	0.559^*^	2.641^*^
ICC municipality	25%	23%	15%	21%
ICC Province	24%	–	12%	35%
N postal code areas	4017	438	1472	2107
N municipalities	396	15	146	235
N provinces	12	–	12	12

^a^Two-level model excluding province due to small number of provinces

^*^p < 0.05

### Ecological correlates of CI’s in all postal code areas

The fixed effects of level of urbanization in the full model for all postal code areas illustrate the spatial concentration of CI’s in urban areas and in (semi-)rural areas and less so in semi-urban and intermediate areas. Population size and the socio-economic status of areas are positively related to the presence of CI’s.

Of the indicators of a care vacuum, only the distance to a high school is associated to the presence of a CI. Of the variables indicating the capacity for concerted action, the percentage of people with Catholic religion is positively associated to the presence of CI’s. The coefficients of social capital and of the percentage of higher educated come close to the conventional significance level of *p* < 0.05. Distance from a postal code area to the nearest area with a CI – as an indicator for the availability of models of action – is significantly related to the presence of CI’s.

The explanatory power of the models is approximated by pseudo R-squared. The fixed effects in the models explain between 24% (for the total dataset) and 63% (for urban postal code areas). Including the systematic variation at municipal and province level, the pseudo R-squared ranges from 31 to 63%.

### Stratified analysis

We ran a separate analysis for level of urbanization in three categories (Table 2). This stratified analysis shows that population density is related to CI’s in areas with a larger population in urban and semi-urban and intermediate areas, but not in (semi-) rural areas. Socio-economic status of the areas is only related to CI’s in urban areas. We found more postal code areas with a CI in urban neighbourhoods with a higher socio-economic status.

**Table 2:** Logistic multilevel analysis with dependent variable presence (1) or absence (0) of a citizen’s initiative; full model for total dataset and stratified by level of urbanization; B coefficient (standard error)

	Total dataset	Urban ^a^	Semi-urban and intermediate	Semi-rural and rural
Constant	−2.24 (.591)	−4.67 (.654)	−3.16 (.387)	−2.73 (.600)
Confounders				
level of urbanization: semi-urban and intermediate (ref = urban)	−1.25 (.504)^*^	–	–	–
level of urbanization: semi-rural and rural (ref = urban)	−.56 (.583)	–	–	–
Population / 10,000	.94 (.267)^*^	2.16 (.593)^*^	1.24 (.470)^*^	.29 (.519)
Socio-economic status	.23 (.119)^*^	.86 (.228)^*^	−.12 (.205)	.06 (.271)
Care vacuum				
Percentage of 75+	.02 (.036)	−.00 (.092)	.01 (.060)	.05 (.075)
Current distance GP	.17 (.139)	−3.48 (1.691)^*^	−.01 (.355)	.14 (.169)
Current distance pharmacy	.066 (.108)	−3.47 (1.845)	−.02 (.240)	.04 (.137)
Current distance hospital	.04 (.028)	.15 (.157)	−.02 (.070)	.07 (.037)
Current distance grocery store	−.27 (.173)	1.62 (1.588)	.19 (.377)	−.34 (.217)
Current distance elementary school	.07 (.299)	−.76 (1.867)	.40 (.695)	.07 (.375)
Current distance high school	.17 (.047)^*^	.09 (.459)	.19 (.130)	.17 (.060)^*^
Current distance library	.02 (.070)	−.11 (.380)	.27 (.155)	−.01 (.094)
Distance to GP increased (ref = no increase)	.17 (.396)	b	.05 (.836)	.83 (.522)
Distance to pharmacy increased (ref = no increase)	.38 (.346)	4.26 (1.706)^*^	.58 (.622)	.45 (.472)
Distance to hospital increased (ref = no increase)	−.19 (.213)	.58 (.438)	−.58 (.414)	−.56 (.374)
Distance to grocery store increased (ref = no increase)	.84 (.480)	b	.22 (1.034)	1.09 (.630)
Distance to elementary school increased (ref = no increase)	−.60 (.634)	b	b	−.19 (.759)
Distance to high school increased (ref = no increase)	−.50 (.260)	−.013 (.602)	−.58 (.594)	−.57 (.378)
Distance to library increased (ref = no increase)	−.16 (.257)	.65 (.550)	−.38 (.482)	−.56 (.456)
Percentage of migrants	.01 (.012)	.06 (.021) ^*^	−.02 (.027)	.01 (.028)
Capacity variables				
Social capital	1.59 (.995)	−.64 (1.913)	1.89 (1.806)	4.69 (1.897)^*^
Percentage 65–75	.012 (.052)	.33 (.159) ^*^	.05 (.084)	−.07 (.089)
Residential turnover	.003 (.006)	−.02 (.010)	.02 (.011)	−.02 (.011)
Percentage high education	.02 (.020)	.15 (.045) ^*^	.08 (.037)^*^	−.02 (.034)
Percentage Catholic	.02 (.008)^*^	−.03 (.053)	.03 (.013)^*^	.02 (.011)^*^
Model of action				
Distance to other initiatives	−.19 (.097)^*^	.66 (.348)	−.10 (.154)	−.18 (.144)
N postal code areas	2387	345	1054	946
N municipalities	377	15	143	219
N provinces	12	–	12	12
Pseudo R-squared (incl. Random effects	0.42	0.63	0.31	0.58
Pseudo R-squared (fixed effects)	0.24	0.63	0.26	0.29

^a^Two-level model excluding province due to small number of provinces

^b^No increase of distance

^*^p < 0.05

Of the care vacuum indicators in urban areas we found significant coefficients for distance to the nearest GP (negative; shorter distance, higher chance of a CI, which is contrary to the hypothesis), when the distance to the nearest pharmacy had increased, and with a higher percentage of people with a migration background in the area. In semi-urban and intermediate areas we found no significant main effects of the care vacuum indicators. In (semi-)rural areas we only found a significant main effect of the distance to the nearest high school: the chance of a CI was higher when the nearest high school is further away.

Of the capacity variables in urban areas we found positive associations with percentage of the population aged 65 to 75 years and with the percentage of highly educated people in the municipality. In the intermediate areas we found positive associations with the percentage of highly educated people in the municipality and with the percentage of the population with catholic religion. In the (semi-)rural areas social capital and the percentage of the population with catholic religion were positively associated with the presence of CI’s.

In the stratified analysis the distance to a postal code area with a CI was not related to the presence of CI’s.

### Interactions

**Fig. 2:**
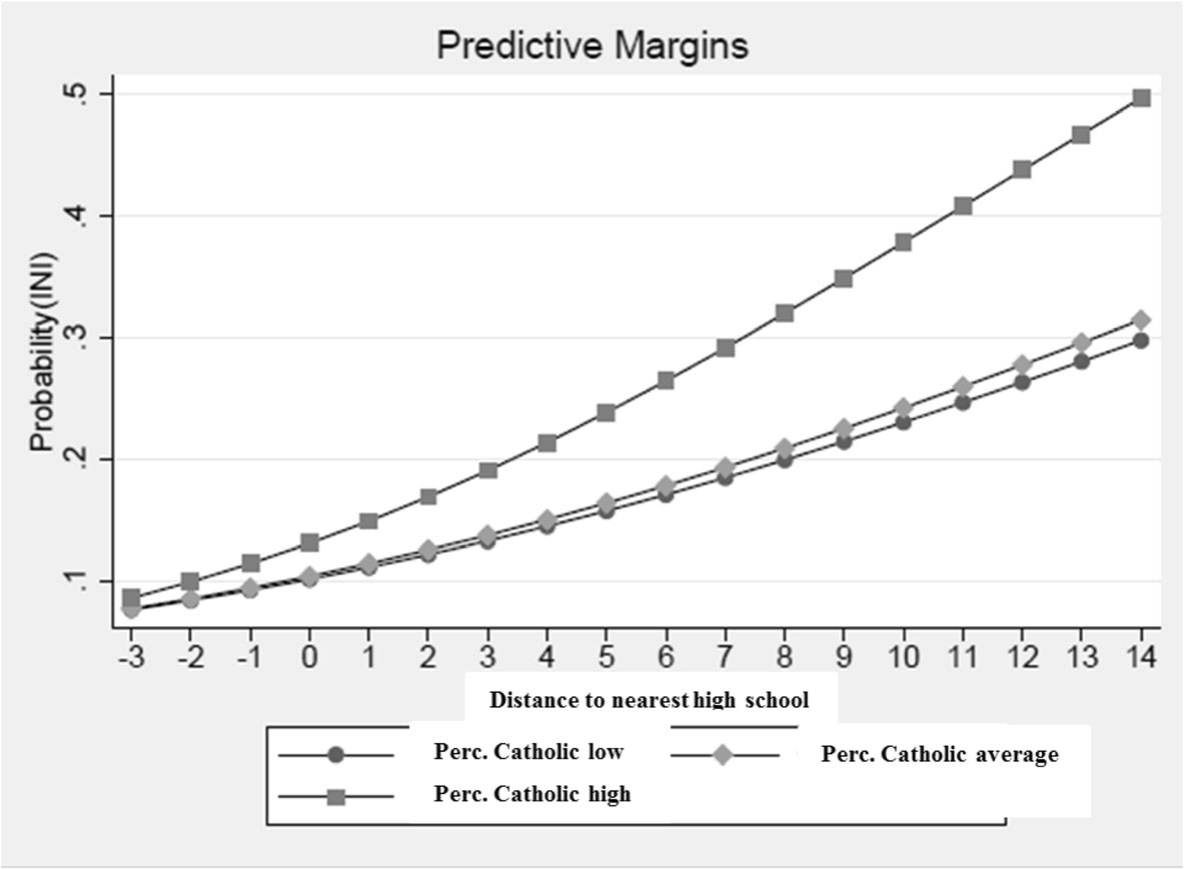
Interaction between the distance to the nearest high school and the percentage of Catholics in the analysis of all postal code areas. Predictive margins, based on the logistic multilevel analysis, presented in Table 4 in Appendix. Reduced model for all postal code areas with interactions, including independent variables only when the standard error was smaller than the regression coefficient. Source: own production

Our theoretical model assumes that the presence of CI’s depends on the combination of a care vacuum and the capacity for concerted action and models of action. We have tested the interactions in a reduced regression model and only for significant main effects. In this approach we found a significant interaction in the analysis of all postal code areas between the distance to the nearest high school – one of the care vacuum indicators - and the percentage of Catholics – one of the capacity indicators. The coefficient of this interaction term is positive, indicating an increased chance of the presence of a CI when both the distance to the nearest high school and the percentage of Catholics are higher in a postal code area (Fig. 2 and Table 4 in Appendix). The second interaction tested for all postal code areas was that between the distance to a high school and the distance to other initiatives. This interaction term was not significant.

None of the interactions estimated in the stratified analyses was significant.

## Discussion

Citizen’s initiatives are unevenly spread over the Netherlands with a strong concentration in the south-east of the country and in three of the biggest cities of the Netherlands which are all located in the Randstad provinces. They are clustered within municipalities and provinces.

An interesting finding is that while CI’s are present in large numbers in three of the biggest cities of the Netherlands (Amsterdam, Den Haag and Utrecht), they are present to a much smaller extent in the second largest city of the Netherlands (Rotterdam). Reasons for this finding cannot be given based on the results of our study. We can speculate that the reason is that Rotterdam scores worse on social and economic indicators, like average education level and income compared to the other cities and that this undermines the capacity to form CI’s.

We tested hypotheses about the effects of a care vacuum, the capacity for concerted action and models of care and interactions between these groups of variables. Our hypotheses were only partly confirmed, as shown in the summary table (Table 3). The indicators for a care vacuum showed some hypothesised relations with the presence of a CI. The distance to the nearest high school – an indicator for a lack of close by services in general rather than of a care services – was related to the presence of a CI in the overall analysis and in rural areas. The percentage of people with a migration background was positively related to presence of a CI in urban areas, controlling for the socio-economic status of areas. Of the variables that indicate the capacity for concerted action we found more of the hypothesised associations. Social capital was related to the presence of CI’s in rural areas, as was the percentage of people with a Catholic religious affiliation. The latter variable was also associated with the presence of CI’s in areas with intermediate level of urbanization and in the overall analysis. The percentage of people with higher education was associated with the presence of initiatives in urban and intermediate areas. The distance to the nearest area with a CI was related to the presence of an initiative only in the overall analysis. Only one of the hypothesised interactions was found; in areas with a higher percentage of people of Catholic religion *and* a longer distance to the nearest high school the chance of a CI was higher.

**Table 3:** Summary table of hypothesised relationships and results of the analyses

Results	All areas	Urban	Semi-urban and intermediate	Semi-rural and rural
Mechanism and variable				
Care vacuum				
Percentage of 75+	Ns	Ns	Ns	Ns
Current distance GP	Ns	–	Ns	Ns
Current distance pharmacy	Ns	Ns	Ns	Ns
Current distance hospital	Ns	Ns	Ns	Ns
Current distance grocery store	Ns	Ns	Ns	Ns
Current distance elementary school	Ns	Ns	Ns	Ns
Current distance high school	+	Ns	Ns	+
Current distance library	Ns	Ns	Ns	Ns
Distance to GP increased	Ns	Ns	Ns	Ns
Distance to pharmacy increased	Ns	+	Ns	Ns
Distance to hospital increased	Ns	Ns	Ns	Ns
Distance to grocery store increased	Ns	Ns	Ns	Ns
Distance to elementary school increased	Ns	Ns	Ns	Ns
Distance to high school increased	Ns	Ns	Ns	Ns
Distance to library increased	Ns	Ns	Ns	Ns
Percentage of migrants	Ns	+	Ns	Ns
Capacity for action				
Social capital	Ns	Ns	Ns	+
Percentage 65–75	Ns	+	Ns	Ns
Residential turnover	Ns	Ns	Ns	Ns
Percentage high education	Ns	+	+	Ns
Percentage Catholic	+	Ns	+	+
Model of action				
Distance to other initiatives	+	Ns	Ns	Ns
Interactions^a^				
Distance to nearest high school and percentage Catholic	+	Ns	Ns	Ns

+ = significant coefficient in hypothesized direction

- = significant effect in opposite direction

Ns = non-significant coefficient

^a^Only one of the tested interactions had a significant coefficient

We conclude that we did not find strong evidence for the theoretical model and hypotheses we have developed. It seems that the capacity for concerted action is the most important mechanism in explaining the presence of a CI. For a feeling of necessity to develop the quality of services may be important, next to the distance to services. The match between services and people’s needs and expectations may also depend on the cultural homogeneity of neighbourhoods. There is an apparent concentration of citizen’s initiatives in the south-eastern provinces of Noord-Brabant and Limburg. These two provinces are traditionally more catholic compared to the protestant majority in the rest of the Netherlands. Underlying cultural differences, related to the religious divide, might also be related to the differences in the presence of citizens initiatives. All three confounders that we included in the analysis were related to the presence of CI’s. Level of urbanization turned out to be especially important, with a higher presence in urban and rural areas compared to intermediate areas; this is why we decided for a stratified analysis. Population density and the socio-economic status of neighbourhoods are related to the presence of CI’s in urban areas, but not in rural areas. One of the indicators for a care vacuum, distance to the nearest GP practice, was unrelated to the presence of CI’s in rural and intermediate areas, but contrary to our hypothesis, negatively related in urban areas. This points to the need to further develop the theoretical model to explain the different situation and perhaps different role of CI’s in urban and rural areas.

Although we could only partly confirm our hypotheses, we know more than before. In particular, we know now that the existence of a care vacuum does perhaps not have a decisive influence on the presence of citizens initiatives. There are several possible reasons for this. At the level of the theoretical model, we have only analysed the implications at community level. We did not have information about the individual level process of experiencing a care vacuum and the subsequent feeling of necessity for action among enough people to make action feasible. Another reason for a lack of significant associations might lay in the definition of the dependent variable. The theoretical model reasons from the establishment of an initiative (the birth of a formal or informal organisation). In the empirical analysis we have looked at the presence of an active CI at one point in time. In this way, we have missed initiatives that have existed for some time, but have ceased to exist.

Quantitative research into citizen’s initiatives in care and welfare is very sparse, especially in the Netherlands. Partly due to this fact we did not find explanatory models and empirical tests of geographical correlates of the presence of CI’s. Davitt et al. [9] mention conditions that might contribute to the establishment of ‘Villages’, a form of CI’s in the USA. Some of these come close to mechanisms in our theoretical model, particularly the idea of policy gaps that might lead to a care vacuum, and the importance of community capacity for self-organisation. Buckingham et al. [7] in their study of the regional distribution of social enterprises in the UK, also mention local needs and capacity, in the form of social capital, but also compensating government support in deprived areas. However, neither of them has systematically worked this out into hypotheses and done an empirical test. This is important, because it teaches us more about mechanisms that may explain the geographical distribution of CI’s; and also when a number of operationalisations in terms of measured variables, do not have the hypothesised effect, we know more than before.

A limitation of this study is that we cannot be sure that our inventory of CI’s is complete. We may have missed e.g. some of the smaller initiatives that are not visible on the internet and that are not members of the national network. We may also have missed initiatives where no regional networks are active and where the visibility of CI’s is less.

We conducted a cross-sectional analysis; hence we could not study the ‘birth’ and ‘death’ of citizen’s initiatives. Also the causal order of the studied relationships could not be assessed. It is possible that starting a CI creates social capital, rather than the other way around [13]. Our operationalisations were restricted by the data that were available at the level of postal code areas or municipalities. In particular, we did not have survey results on the experience of a care vacuum, nor about other models of action, such as the density of clubs and associations. A further limitation is that we did not use data about changes in the distance to typical elderly care facilities, such as homes for the elderly and nursing homes.

The study also has strengths. The unique character of this study is that it provides a theory-based, empirical test of hypotheses. Moreover, it is not based on case studies but on the best available information for the whole of the country.

## Conclusion

There are by now a considerable number of CI’s in the area of care and welfare in the Netherlands. Apparently, citizens take collective initiatives to provide care and welfare services to the local community. The initiatives are concentrated in certain parts of the country. Since January 2016, the reference date for our data, the number of citizen initiatives has increased fast. Nederland Zorgt Voor Elkaar, the national network of CI’s in care and welfare, now estimates their number to be over 800. National support and regional networks of CI’s may have been helpful.

For future research four directions are important. The first is to follow CI’s longitudinally; this may also provide an opportunity to link the emergence of CI’s to local differences in care and welfare policies by municipalities, as a result of the devolvement of responsibilities. The second is to include the level of individual citizens e.g. by surveying the perception of an unwanted situation and the willingness to take action. The third is to further develop the theoretical model and mechanisms to include cultural differences between areas where a lot of CI’s are present and areas where they are not present. These differences might contribute to the explanation of the geographical pattern that we found.

The fourth is to differentiate between different types of CI’s, e.g. CI’s that provide only welfare and those that provide both welfare and care or CI’s that provide combinations of care and welfare and other services. Different types of CI’s might differ from each other in their typical locations (e.g. urban vs. rural) or the process of getting established.

## Data Availability

The datasets used and/or analysed during the current study are available from the corresponding author on reasonable request.

## Appendix

**Table 4:** Logistic multilevel analysis with dependent variable presence (1) or absence (0) of a citizen’s initiative; reduced model for all postal code areas with interactions; B coefficient (standard error)

	B coefficient
Constant	−2.28 (.555)
Confounders	
Level of urbanization: semi-urban and intermediate (ref = urban)	−1.29 (.473)^*^
Level of urbanization: semi-rural and rural (ref = urban)	−.61 (.545)
Population / 10,000	.94 (.257)^*^
Socio-economic status	.21 (.105)^*^
Care vacuum	
Current distance GP	.23 (.111)^*^
Current distance hospital	.02 (.025)
Current distance grocery store	−.24 (.168)
Current distance high school	.12 (.050)^*^
Distance to pharmacy increased (ref = no increase)	.45 (.274)
Distance to grocery store increased (ref = no increase)	.72 (.452)
Distance to high school increased (ref = no increase)	−.54 (.256)^*^
Percentage of migrants	.01 (.012)
Capacity variables	
Social capital	1.65 (.984)
Percentage Catholic	.02 (.008)^*^
Model of action	
Distance to other initiatives	−.23 (.095)^*^
Interaction of distance to high school and distance to other initiatives	−.04 (.026)
Interaction of distance to high school and percentage Catholic	.003 (.001)^*^

^*^*p* < 0.05
